# Identification of ribosome biogenesis genes and subgroups in ischaemic stroke

**DOI:** 10.3389/fimmu.2024.1449158

**Published:** 2024-09-02

**Authors:** Xi Wang, Xiao-Yu Zhang, Nan-Qing Liao, Ze-Hua He, Qing-Feng Chen

**Affiliations:** ^1^ School of Medicine, Guangxi University, Nanning, China; ^2^ The College of Life Sciences, Northwest University, Xian, China; ^3^ Department of General Surgery, Guangxi Hospital Division of The First Affiliated Hospital, Sun Yat-sen University, Nanning, China; ^4^ School of Computer, Electronics and Information, Guangxi University, Nanning, China

**Keywords:** ribosome biogenesis, stroke, CIBERSORT, immune infiltration, neutrophil

## Abstract

**Background:**

Ischaemic stroke is a leading cause of death and severe disability worldwide. Given the importance of protein synthesis in the inflammatory response and neuronal repair and regeneration after stroke, and that proteins are acquired by ribosomal translation of mRNA, it has been theorised that ribosome biogenesis may have an impact on promoting and facilitating recovery after stroke. However, the relationship between stroke and ribosome biogenesis has not been investigated.

**Methods:**

In the present study, a ribosome biogenesis gene signature (RSG) was developed using Cox and least absolute shrinkage and selection operator (LASSO) analysis. We classified ischaemic stroke patients into high-risk and low-risk groups using the obtained relevant genes, and further elucidated the immune infiltration of the disease using ssGSEA, which clarified the close relationship between ischaemic stroke and immune subgroups. The concentration of related proteins in the serum of stroke patients was determined by ELISA, and the patients were divided into groups to evaluate the effect of the ribosome biogenesis gene on patients. Through bioinformatics analysis, we identified potential IS-RSGs and explored future therapeutic targets, thereby facilitating the development of more effective therapeutic strategies and novel drugs against potential therapeutic targets in ischaemic stroke.

**Results:**

We obtained a set of 12 ribosome biogenesis-related genes (EXOSC5, MRPS11, MRPS7, RNASEL, RPF1, RPS28, C1QBP, GAR1, GRWD1, PELP1, UTP, ERI3), which play a key role in assessing the prognostic risk of ischaemic stroke. Importantly, risk grouping using ribosome biogenesis-related genes was also closely associated with important signaling pathways in stroke. ELISA detected the expression of C1QBP, RPS28 and RNASEL proteins in stroke patients, and the proportion of neutrophils was significantly increased in the high-risk group.

**Conclusions:**

The present study demonstrates the involvement of ribosomal biogenesis genes in the pathogenesis of ischaemic stroke, providing novel insights into the underlying pathogenic mechanisms and potential therapeutic strategies for ischaemic stroke.

## Introduction

Stroke is a group of diseases with symptoms of ischemic and hemorrhagic damage to the brain as the main clinical manifestation, including ischemic stroke and hemorrhagic stroke (1). Among them, ischemic stroke (IS) accounts for about 80% of all stroke cases, occurring in middle-aged and elderly people, and is characterized by high mortality and disability rates. Its treatment and management have been viewed as a serious medical and public health challenge (2).

Ischemic stroke is primarily caused by a sudden disruption of blood flow due to thrombosis or embolism. The IS pathological process involves a complex temporal and spatial cascade of responses and is the result of multiple cellular pathways. Ischemia caused by stroke restricts blood flow to specific regions of the brain, inducing a series of pathological reactions that culminate in the infiltration of immune cells (3, 4). Furthermore, the immune microenvironment and inflammatory response play a crucial role in the development of IS and are closely linked to the severity and prognosis of the condition (5). Thus, the discovery of new therapeutic targets for IS can be facilitated by gaining a deeper understanding of stroke pathogenesis and immune microenvironment changes, which may offer fresh prospects and avenues for exploration. In general, the standard pathological change after the onset of IS is the disruption of the blood-brain barrier, leading to the gathering of various infiltrating immune cells, such as T cells, B cells, neutrophils, dendritic cells, and macrophages, in the edema area (6). These immune cells play a dual role (7). Also, immune cells release a significant number of inflammatory cytokines, which can promote secondary neuroinflammation. Many immune cells remove necrotic cell debris, reduce the inflammatory response and play a protective role in repairing the blood-brain barrier and promoting angiogenesis (8). Immunomodulation after stroke has therefore become an important area of research and therapy, aiming to reduce damage and promote recovery by controlling the intensity and nature of the immune response.

Ribosome biogenesis, also known as RiboSis, is a highly intricate process responsible for producing the ribosomes necessary for protein synthesis during various cellular processes such as proliferation, differentiation, apoptosis, development, and transformation (9–12). In stroke, especially in ischaemic stroke, changes in RiboSis may have an important impact on cell fate and disease progression. Further, after cerebral ischaemia, cells undergo metabolic reprogramming to adapt to changes in energy demand (13). This metabolic adjustment affects multiple steps in ribosome biogenesis. Ribosomes are a common component of autophagy. After a stroke, cells may break down existing ribosomes and initiate autophagy mechanisms to recycle damaged cellular components and gain energy (14, 15). Ribosomes are not only the site of conventional protein synthesis, but are also involved in the synthesis of certain neuroprotective proteins such as growth factors and antioxidant proteins. For example, certain neuroprotective strategies may enhance neuronal resistance to ischaemic injury or accelerate its recovery by promoting the expression of specific ribosomal proteins or modulating certain steps in ribosome biogenesis. Further, as mentioned earlier, the inflammatory response to stroke involves the synthesis of a large number of immune-related proteins, and their production is directly dependent on ribosome function. Precision medicine has become the main development trend in the clinical treatment of many chronic diseases, especially the accurate classification and hierarchical management of patients. Given the significance of Ribosis in the cellular response to stroke, to construct a classification model of Ribosis in the cellular response to stroke, and to study its regulatory mechanism may find new therapeutic methods. Previous studies have reported that ribosomal biogenesis plays a central role in cancer (16, 17). Ribosome biogenetic risk scores have been reported in cancer (18). To date, few classification models constructed with ribosome biogenesis-related genes have been applied to stroke research.

**Figure 1:**
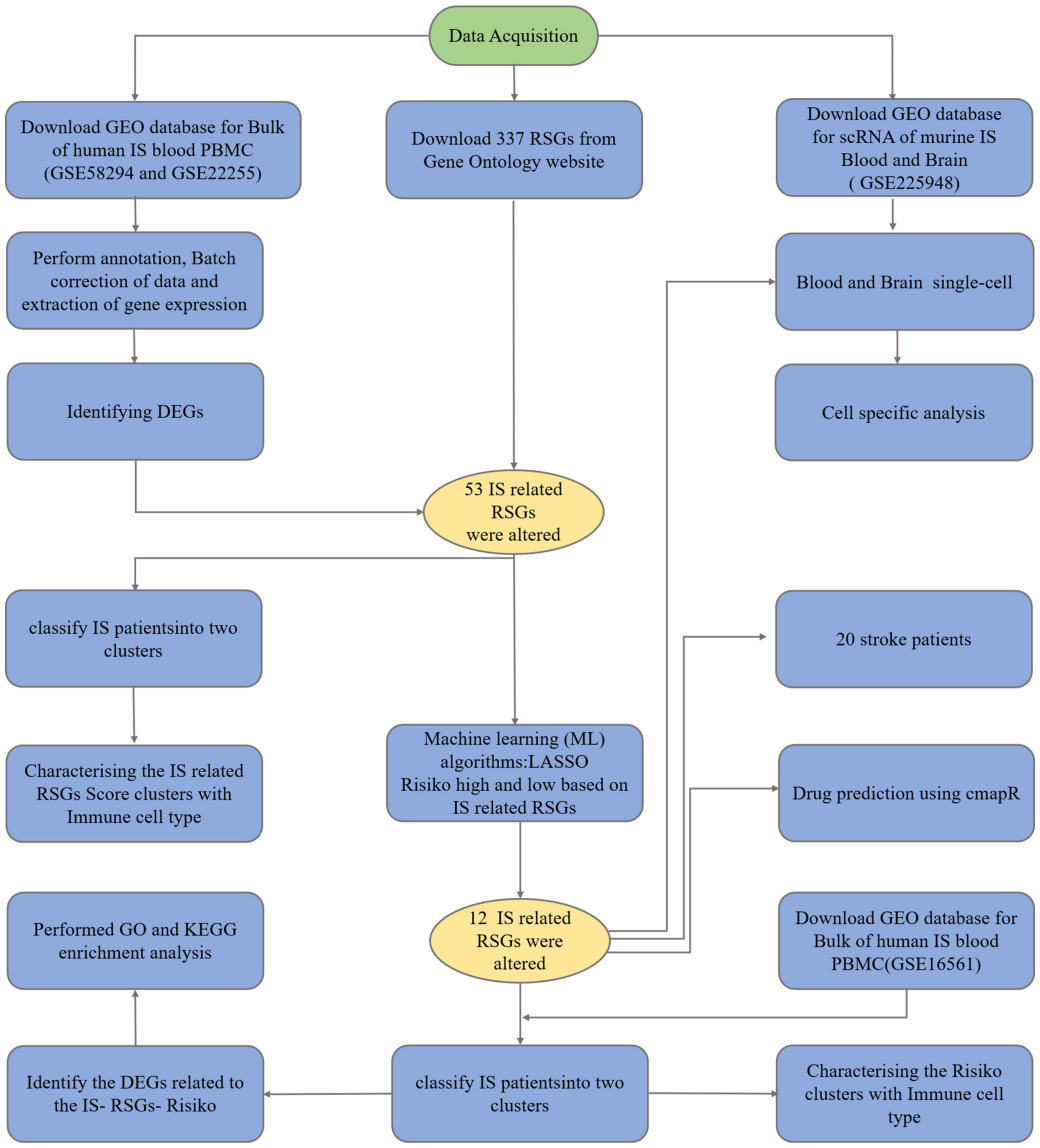
Research flowchart.

As a member of the predictive modelling and data analysis toolbox:lasso regression, this technique is appreciated for its accuracy and versatility in various fields, and is also widely used in medicine (19). As part of linear regression, Lasso regression addresses the infamous “curse of dimensionality”, where the number of predictors exceeds the observed value (20, 21). It contains penalties for large coefficients to simplify the model and keep it comprehensibility (21). It has been used to construct classification models in diseases such as cervical cancer and breast cancer (22–24). Our study can be briefly described by **Figure 1**, in which we constructed a high-risk group model with genes related to ribosomal biogenesis and assessed the immune infiltration of IS patients by the CIBERSORT method. We also analysed the characteristics of observed immune cell subtype specificity by scRNA sequencing. In conclusion, we used ribosome biogenesis-related genes to classify and observe immune cell infiltration in stroke patients. The proposed strategy may provide further ideas for future clinical studies on prevention and treatment of high and low risk groups. It is expected to provide a unique perspective for exploring new ways of stroke prevention or treatment.

## Materials and methods

### Datasets and quality control

The RNA-seq gene expression files was obtained from Gene Expression Omnibus (GEO) database, which can be accessed at (GEO, https://www.ncbi.nlm.nih.gov/geo/) GSE58294 (25)(containing 20 Control and 69 Stroke samples), GSE22255 (26)(containing 20 Control and 20 Stroke samples) GSE16561 (27)(containing 24 Control and 39 Stroke samples).

All statistical analyses and graphical representations in this study were conducted using the R 4.2.2 software. During the data preprocessing stage, probes without corresponding genes were removed, and the average was taken when multiple probes corresponded to one gene. Standardization of the data was performed, followed by merging the two series. The limma package in R was utilized to standardize the data and eliminate the batch effect. It is important to note that this research adhered to the data access rules and release principles of the database in question.

### Ribosome biogenetic genes related gene collection

We obtained the seven ribosome biological genetic related pathways from the GO database (https://www.geneontology.org/) (RIBOSOME BIOGENESIS, RRNA TRANSCRIPTION, obsolete cleavage involved in rRNA processing, RRNA MODIFICATION,RIBOSOME ASSEMBLY,RIBOSOMAL SMALL SUBUNIT EXPORT FROM NUCLEUS, RIBOSOMAL LARGE SUBUNIT EXPORT FROM NUCLEUS). 337 ribosome biogenetic genes were eventually sorted out after removing duplicate genes.

### Identification of IS-RSGs in stroke

The DESeq2 package was utilized to identify differentially expressed genes (DEGs) between control samples and stroke samples based on the following criteria: log2(Fold-Change) > 0.232 and adjusted p-value < 0.001. Heatmaps and volcano plots of the differential analysis results were generated using the pheatmap and ggplot2 packages, respectively. The analysis involved the examination of the intersection of differentially expressed genes and RSGs. Subsequently, ssGSEA scores were conducted using GSVA packaging, and patients were classified into high and low score groups based on the median score.

### Estimation of immune cells

The CIBERSORT algorithm (Stanford University’s <https://cibersortx.stanford.edu/>) was employed to determine the proportion of 22 immune cell types in GC samples, using the relative mode and 1000 permutations. Specifically, we utilized CIBERSORT R script v1.04 for the immune infiltration analysis of the samples. The comparison between the composition of immune cells in the high-score and low-score ischemic stroke groups was made. Additionally, the comparison between the immune cells from ischemic stroke patients in the high-risk and low-risk groups was also carried out. The box diagram shows the results obtained through the ggplot2 package in R.

### Gene ontology and Kyoto encyclopedia of genes and genomes analysis

Functional enrichment analysis, including biological processes (BP), cellular compositions (CC), and molecular functions (MF), was conducted for IS-RSGs characteristics using the clusterProfiler package in R. We utilized this package to examine the functional enrichment of the clusterProfiler in KEGG pathways.

### Machine learning to identify RSG biomarkers

Looking at the intersection of differential genes and RSGs. The best prognostic model was obtained by utilizing the lasso Cox technique in the glmnet package with a ten-fold cross-validation set. The prognostic risk model was determined by multiplying all risk factors and factor-related coefficients. The patient cohort was then divided into high-risk and low-risk groups based on the optimal cut-off value calculated by the maxstat package. coef is in **Supplementary Table 1**.


$${\text{R}\text{i}\text{s}\text{k}\text{S}\text{c}\text{o}\text{r}\text{e}=\sum }_{i=1}^{N}[G_{i}*coef_{i}]$$

G=Gene expression,N=11

### Single-cell data download and analysis

GSE225948 and conduct the analysis using the Seurat (28). Principal component analysis (PCA) with 10 principal components (PCs) selected and visualized through Uniform Manifold Approximation and Projection (UMAP). The expression of IS-RSGs gene in different clusters was analysed by cluster dimensionality reduction, and different subsets of cells were labelled by their unique marker genes.

### RNASEL,RPS28 and C1QBP1 protein expression by ELISA

100 μL of serum was added into the micropores pre-coated with the trapped antibodies (RNASEL,RPS28 and C1QBP1), covered with the sealing plate membrane and incubated for 1 hour at 37°C. The serum was then discarded, biotinylated antibody was added to 100 μL per well, and the sealing plate was covered at 37°C for 1 hour. After the liquid is discarded, add 300 μL 1X washing solution to each well and let it stand for 1 minute. Then shake off the washing solution and pat dry on absorbent paper. Repeat this for 3 times. Add 100 μL enzyme conjugate working liquid, cover the sealing plate and incubate for 30 minutes at 37°C After discarding the liquid, the plate was washed for 5 times, and 90 μL substrate was added to the plate for 15 minutes at 37°C away from light. After taking out the specimen plate, 50 μL was added to each well, and the OD value was measured at 450nm wavelength immediately on the Thermo Fischer Multiscan Go Reader. The concentrations of RNASEL,RPS28 and C1QBP1 in the sample were determined by the standard curve of recombinant protein production.

### Statistical analysis and visualization of results: R packages and significance assessment

This study carried out statistical analyses and generated visual plots using R (version 4.2.2), with the help of the ggplot2 package for visualization purposes. (version 3.3.6). Additionally, Seurat analysis was utilized with the Seurat package (version 5.0.1), while UMAP (Uniform Manifold Approximation and Projection) analysis utilized the umap package (version 0.2.7.0). PCA (Principal Component Analysis) analysis was conducted using the R package stats (version 3.6.0). The Wilcoxon rank sum test was used to evaluate discrepancies between groups, with a statistical significance level set at p < 0.05. Patients’ baseline data were analysed using Fisher’s exact test. The p-values associated with this test were *p < 0.05, **p < 0.01, ***p < 0.001, and ****p < 0.0001.

## Results

### Biogenetic identification of differences in ribosome expression between Ischemic stroke and normal subjects and assessment of subgroup of immune cell infiltration

**Figure 2:**
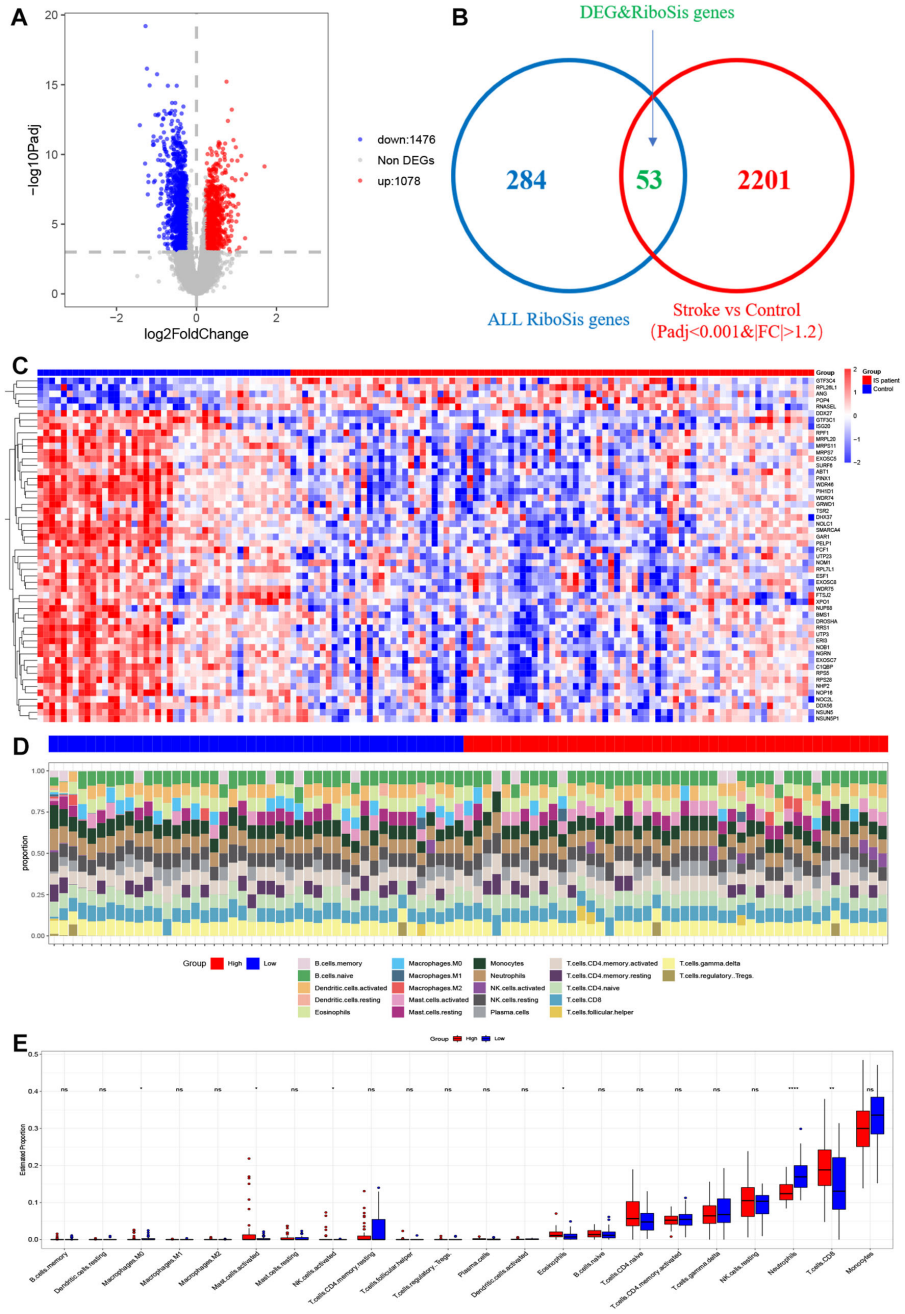
Visualization of deg and DEGS & RSGS in data sets and immune cell infiltration in IS high-score and IS low-score groups **(A)** Volcano plot of DEGs in GSE58294 and GSE16561. **(B)** Venn diagram of common genes between DEGs and ribosome biogenesis genes. **(C)** Heatmap for DEGs&RSGs in Stroke dataset. Red represents high gene expression and blue represents low expression. **(D)** The relative content of 22 kinds of immune cells high score stroke patient group and low score stroke patient group were showed in the histograms. **(E)** The BarPlot illustrated the difference in immune cell infiltration between high score stroke patient group and low score stroke patient group. ns(no significance) p≥0.05; * p< 0.05; ** p< 0.01; **** p< 0.0001.

Ribosome biogenesis is the process by which ribosomes are produced and plays an important role in cell proliferation, differentiation, apoptosis, development and transformation. Therefore, it is urgent to know which ribosome biogenesis genes are differentially expressed between Ischemic stroke and normal samples. We obtained 89 Ischemic stroke samples and 40 normal controls after normalizing the data derived from 2 published Ischemic strokes. We performed differential gene counting on the expression profiles and obtained 2,554 differential genes, including 1,078 up-regulated genes and 1,476 down-regulated genes. DEGs results are presented in the form of volcano plots (**Figure 2A**). By intersecting 2554 DEGs with 337 ribosomal biogenesis genes obtained in GO, 53 key genes were finally identified (**Figure 2B**). We found in the heat map (**Figure 2C**) that most of these genes were highly expressed in normal tissues. We utilized the ssgsea algorithm for immune infiltration analysis to explore differences in immune system between low- and high-score subgroups, revealing disparities in the proportions of 22 infiltrating immune cell types. (**Figure 2D**). The differences in immune cell content between Ischemic stroke and healthy individuals were further explored (**Figure 2E**). The proportion of neutrophils was found to be significantly higher in the low group than in the high group, whereas the proportion of Eosinophils, CD8^+^T cells, NK cells activated, Mast cells activated and Macrophages M0 were higher in the high-score group.

### Identification of RSG signalling constructs and immune cell infiltration in high- and low-risk populations by machine learning

**Figure 3:**
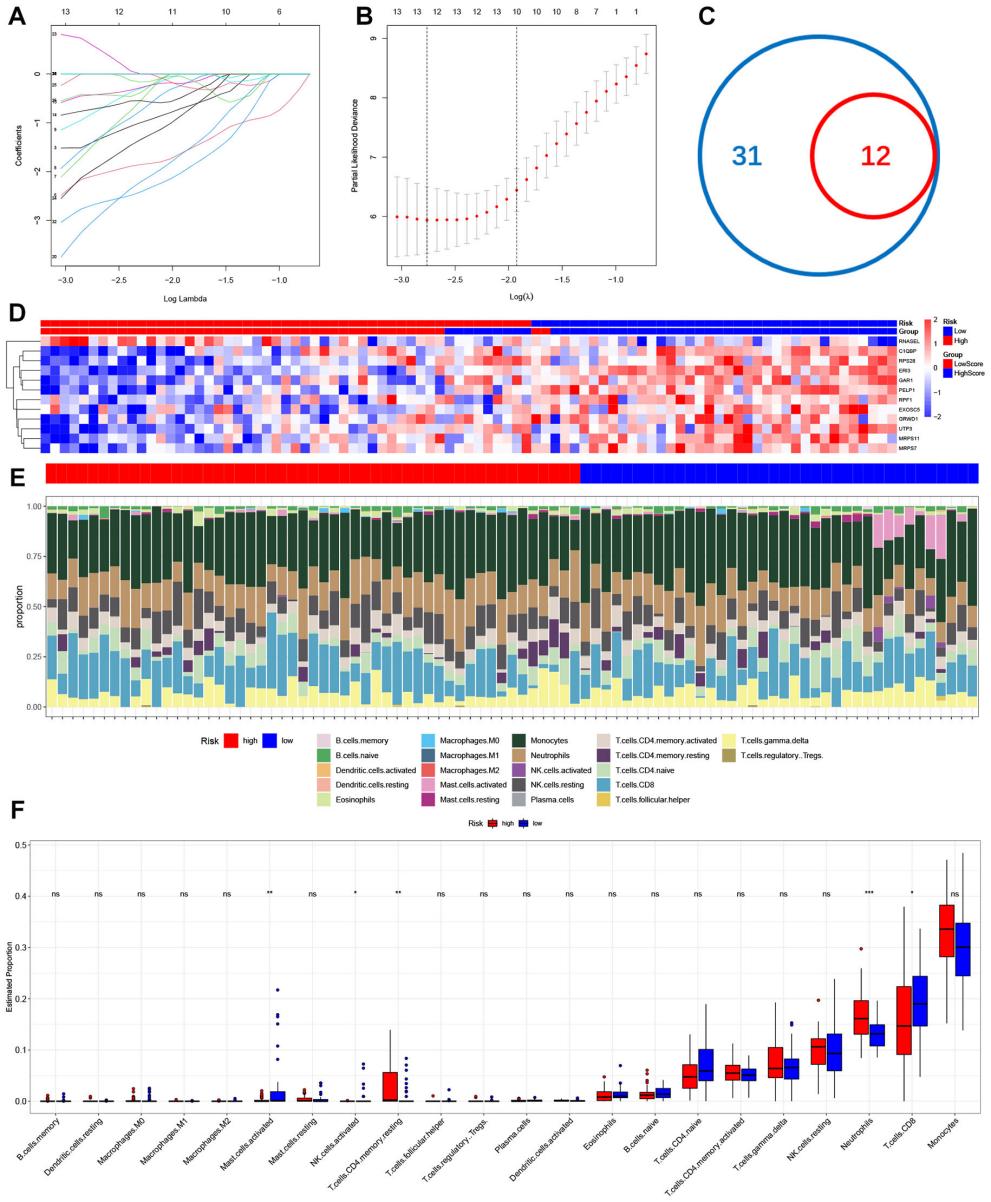
Construction of ribosome biogenic risk signal and infiltration of immune cells in high-risk groups and low-risk groups. **(A, B)** The process of Lasso regression analysis. **(C)** Venn diagram of common genes between DEGs&RSGs and hub genes. **(D)** Heatmap for hub genes in Stroke dataset. **(E)** The relative content of 22 kinds of immune cells high risk stroke patient group and low risk stroke patient group were showed in the histograms. **(F)** The BarPlot illustrated the difference in immune cell infiltration between high risk stroke patient group and low risk stroke patient group. ns(no significance) p≥0.05; * p< 0.05; ** p< 0.01; *** p< 0.001.

To replace our scoring model with fewer genes, we used LASSO Cox regression to build a ribosome biogenesis genes-related feature (RSG) named ribosome biogenesis genes score (**Figures 3A, B**). We ultimately selected 12 genes to calculate the score for each individual (**Figure 3D**) and observed the expression of these 12 genes in the Ischemic stroke samples (**Figure 3D**). Subsequently, we calculated IS-RSGs scores for each individual, allowing us to categorize all individuals into high or low risk categories based on median IS-RSGs scores. Ssgsea algorithm is applied to examine immune infiltration, and our findings revealed that the percentage of 22 distinct immune cell types varied between high-risk and low-risk groups (**Figure 3E**). The individuals in the high-risk group exhibited a significantly higher ratio of neutrophils to T-cell CD4 memory cells compared to those in the low-risk group. Conversely, the low-risk group showed a higher proportion of activated CD8^+^T cells, NK cells, and Mast cells (**Figure 3F**). At the same time, we downloaded GSE16561 for grouping and divided them into two sub-groups using our scoring method. We also used the ssgsea algorithm to analyse the proportion difference of 22 infiltrating immune cell types (**Supplementary Figure 1A**). To further investigate the difference of immune cell content between ischemic stroke and healthy individuals (**Supplementary Figure 1B**). It was also found that the proportion of neutrophils in the low-risk group was significantly higher than that in the high-risk group.

### Differential expression analysis and functional enrichment analysis between risk types

**Figure 4:**
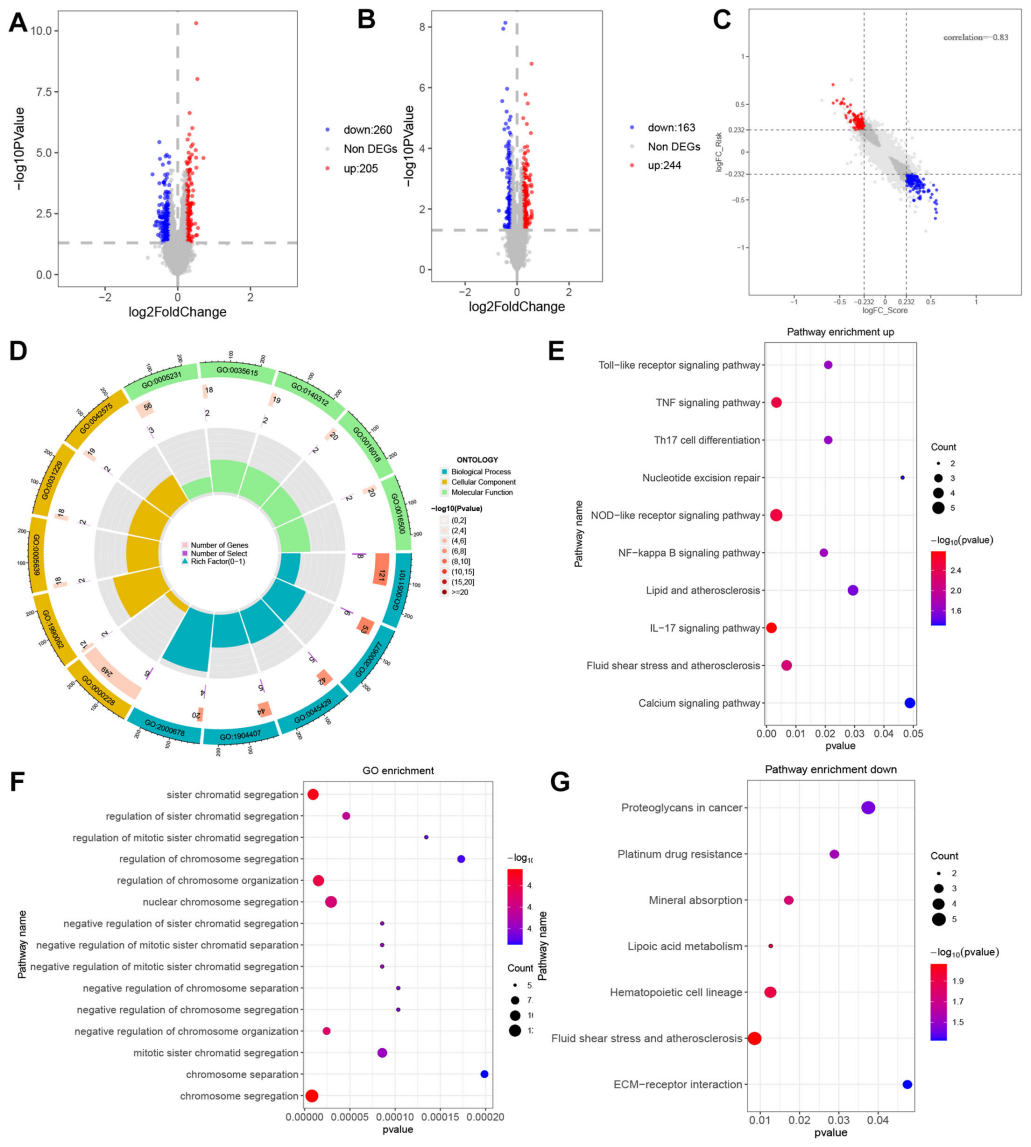
Differential expression analysis and functional enrichment analysis between RiskTypes. **(A)** Volcano plot of differentially expressed genes between high- and low-risk groups; the red dots represent upregulated genes in the high-risk group. **(B)** Volcano plot of differentially expressed genes between high- and low-score groups; the red dots represent upregulated genes in the high-risk group. **(C)** Scatter plot of 9-quadrant associate analyses of DEGs from log2 FC Scoretypes and RiskTypes. **(D)** GO functional enrichment analysis of up DEGs. **(E)** KEGG pathway analysis of up DEGs. **(F)** GO functional enrichment analysis of down DEGs. **(G)** KEGG pathway analysis of down DEGs.

To further understand the differences between these two subgroups, our analysis involved conducting a differential expression study, which resulted in the identification of 465 DEGs, comprising 260 up-regulated and 205 down-regulated genes. The distribution of these genes is depicted in the volcano plot (**Figure 4A**). Compared with the differential genes obtained from the previous high and low DEG groupings (**Figure 4B**) had a significant correlation coefficient of -0.83 (**Figure 4C**). The next step was to analyse the DEGs for their GO functional annotation and KEGG pathway enrichment. The GO analysis (BP) revealed gene enrichment in regulation of DNA binding, regulation of transcription regulatory region DNA binding, positive regulation of nitric oxide biosynthetic process, positive regulation of nitric oxide metabolic process, negative regulation of transcription regulatory region DNA binding (CC) shows gene clustering in nuclear chromosome,RPAP3/R2TP/prefoldin-like complex, integral component of nuclear inner membrane, intrinsic component of nuclear inner membrane, DNA polymerase complex (MF) shows excitatory extracellular ligand-gated ion channel activity, clathrin adaptor activity, cargo adaptor activity, cyclosporin A binding protein-hormone receptor activity (**Figure 4D**). KEGG enrichment analysis showed that these genes were found to be predominantly enriched in genes such as IL-17 signalling pathway, NOD-like receptor signalling pathway, TNF signalling pathway, Fluid shear stress and atherosclerosis, NF-kappa B signalling pathway, Toll-like receptor signalling pathway, Th17 cell differentiation, Lipid and atherosclerosis, Nucleotide excision repair, Calcium signalling pathway (**Figure 4E**). GO analysis of down-regulated genes revealed that the functions of chromosome segregation, sister chromatid segregation, regulation of chromosome organization, negative regulation of chromosome organization, negative regulation of chromosome organization, and nuclear chromosome segregation were annotated. KEGG enrichment analysis showed that these down-regulated genes were mainly enriched for Fluid shear stress and atherosclerosis, Hematopoietic cell lineage, Lipoic acid metabolism, Mineral absorption, Platinum drug resistance, Proteoglycans in cancer and ECM-receptor interaction (**Figure 4G**).

### The expression specificity analysis of hub genes in single cells

**Figure 5:**
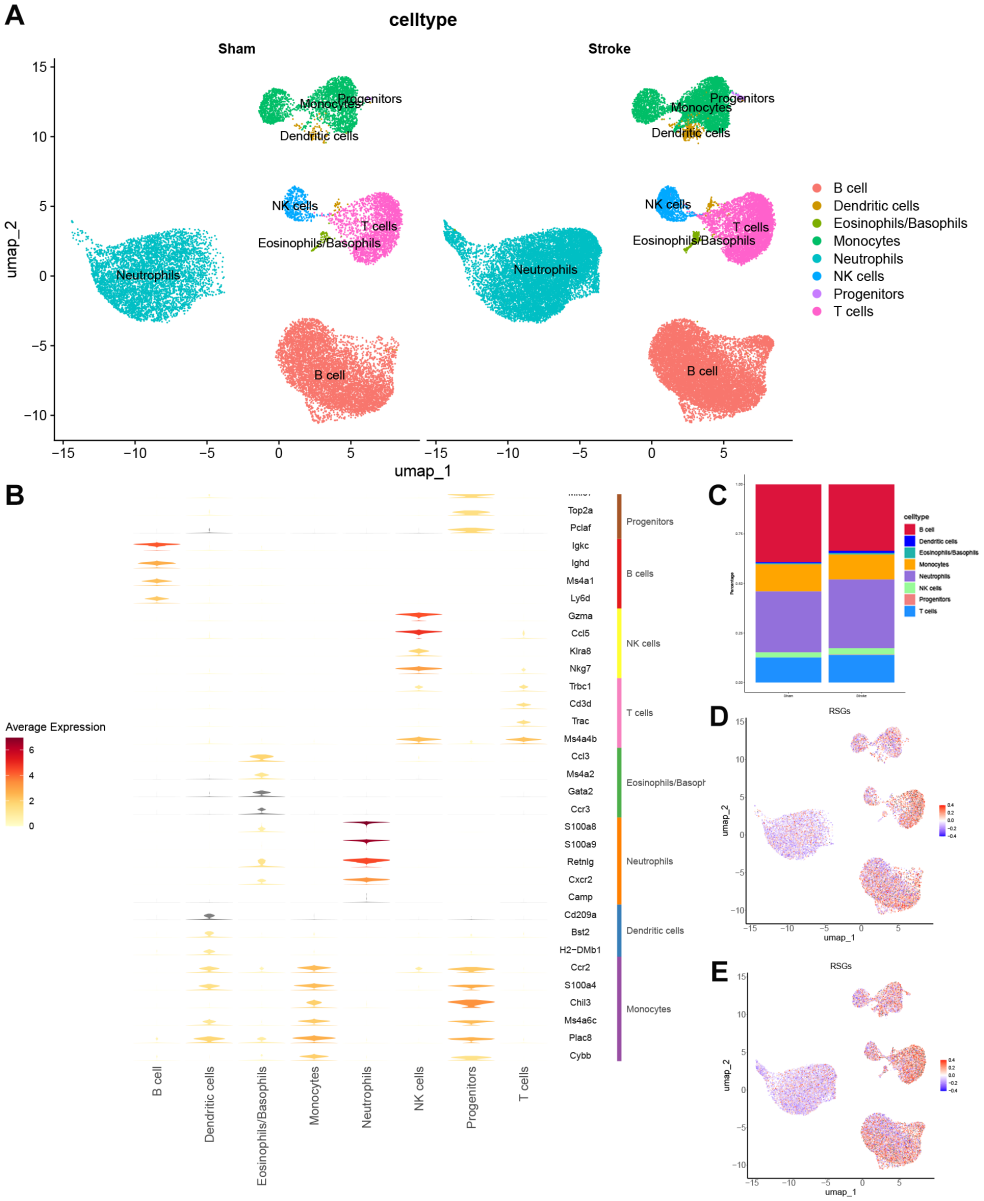
The expression specificity analysis of hub genes in single cells. **(A)** Cell Type Reduction Map Display (UMAP). **(B)** Violin map of marer genes in different cell types. **(C)** Stacked bar chart shows the proportion of different clusters within each group. **(D)** UMAP showed the expression of ribosome biogenesis-related genes set in the all cells subset of Sham. **(E)** UMAP showed the expression of ribosome biogenesis-related genes set in the all cells subset of Stroke.

The analysis of scRNA-seq data included 4 Ischemic stroke samples and 7 sham surgery samples. Following quality filtering, a total of 51,509 cells were screened. Subsequently, the top 10 principal components were selected through principal component analysis. The neighbourhoods between cells were then calculated at a resolution of 0.7, followed by downscaling and projection using UMAP. UMAP downscaling showed that these cells were categorized into 17 clusters (**Supplementary Figure 2A**) These 17 cell clusters expressed markers for 8 known cell types as shown (**Supplementary Figure 2B**). These 17 cell clusters were further divided into 8 known cell lineages (**Supplementary Figure 2C**), Distribution and cell proportion of 8 known cell types in sham operation and Ischemic stroke (**Figure 5A**). Marker expression of these 8 cell types (**Figure 5B**) and their proportion in Sham and Ischemic stroke (**Figure 5C**). We observed the IS-RSGs gene set score in all cells, Neutrophils in both the sham and ischemic stroke groups had lower scores (**Figures 5D, E**).

### Expression specificity analysis of hub genes in neutrophil cells

**Figure 6:**
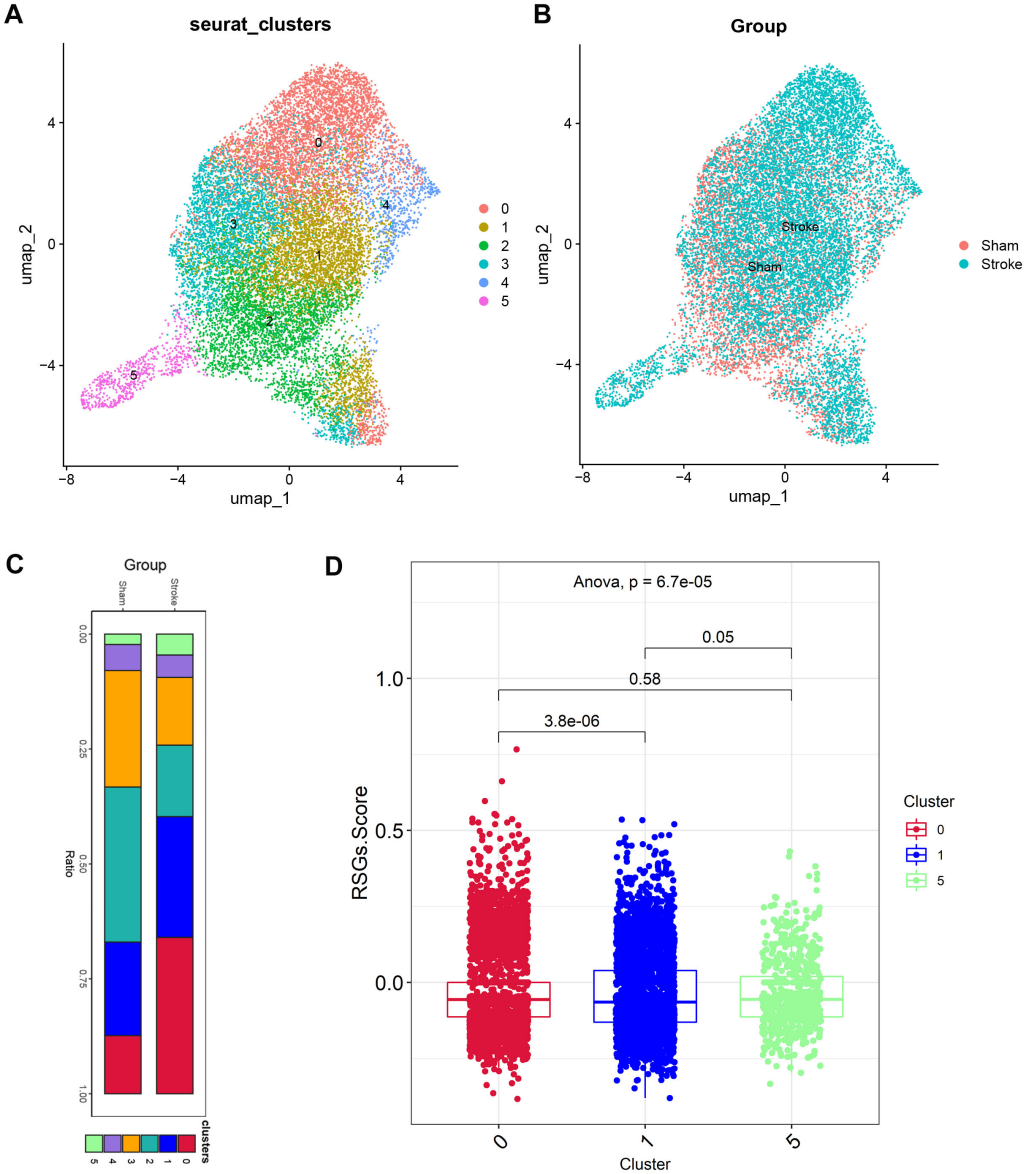
The expression specificity analysis of hub genes in Neutrophil cells. **(A)** Dimensionality reduction map of Neutrophil cell subsets. **(B)** Group display of Neutrophil cell subpopulation type Reduction map (UMAP). **(C)** Stacked bar chart shows the proportion of different clusters within each group. **(D)**The boxplot shows the representation of RSGs Score in the three Clusters.

In earlier times, we discovered disparities in neutrophils among the high-risk group. We subsequently isolated a total of 17,181 cells from the neutrophils subgroup and selected the top ten principal components following principal component analysis. The neighbourhood relationships between cells were calculated at a resolution of 0.3, followed by downscaling and projection using UMAP. The UMAP downscaling revealed that these cells were grouped into 6 clusters (**Figure 6A**). In **Figures 6B, C**, we found an increase in neutrophils in cluster 0, cluster 1, cluster 5 in the ischemic stroke group compared to the sham operation group. The IS-RSGs score of Cluster1 in neutrophils was lower than that of cluster0 and Cluster5 (**Figure 6D**, **Supplementary Figure 2D**).

### Expression specificity analysis of hub genes in CD4^+^T cells and CD8^+^T cells

**Figure 7:**
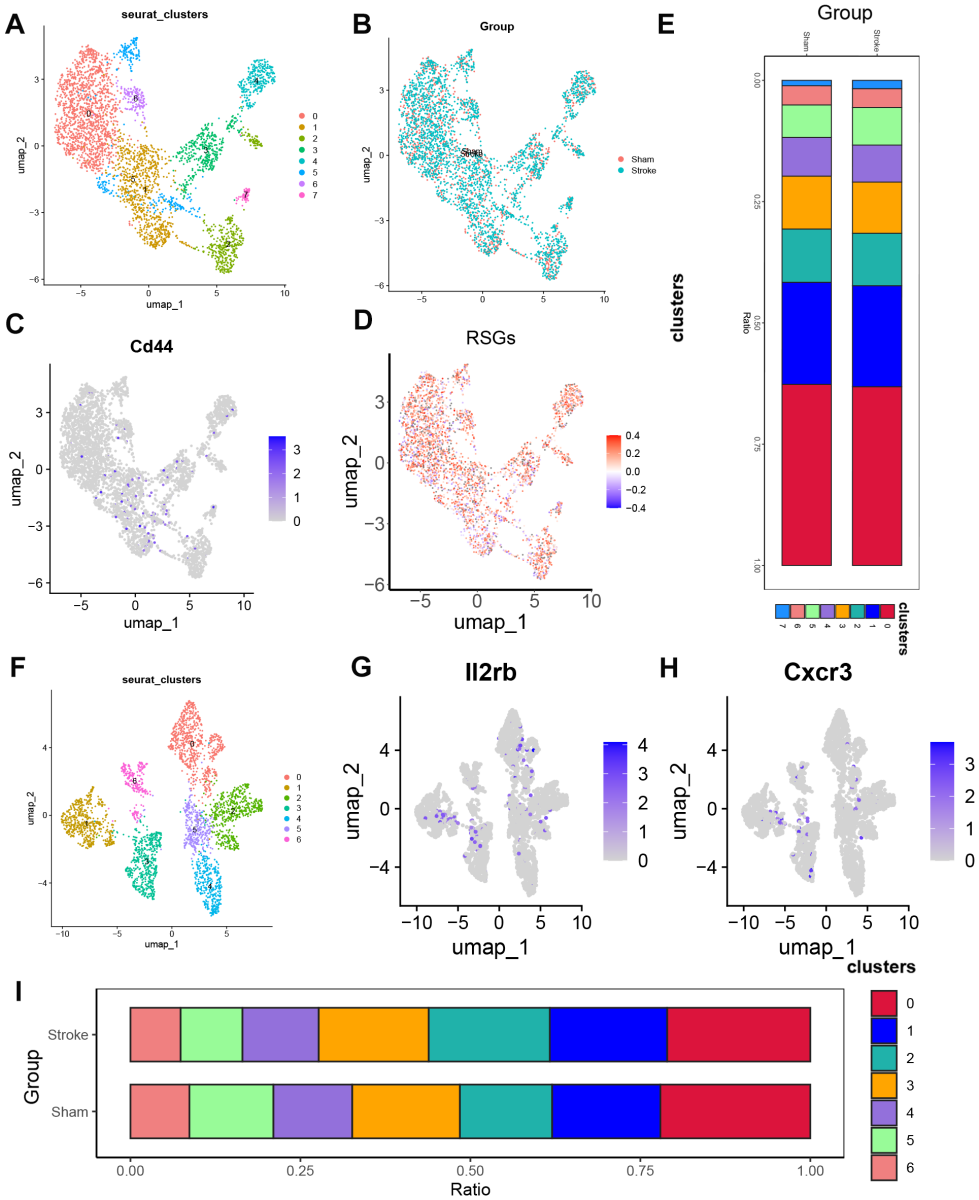
The expression specificity analysis of hub genes in T cells. **(A)** Dimensionality reduction map of CD4 T cell subsets. **(B)** Group display of CD4 T cell subpopulation type Reduction map (UMAP). **(C)** UMAP shows the annotation of Cd44 in Cd4 cells. **(D)** UMAP showed the expression of ribosome biogenesis-related genes set in the CD4 T cells subset of patients. **(E)** Stacked bar chart shows the proportion of different groups within each cluster. **(F)** Dimensionality reduction map of CD8 T cell subsets. **(G)** UMAP shows the annotation of Il2rb in CD8 T cells. **(H)** UMAP shows the annotation of Cxcr3 in CD8 T cells. **(I)** Stacked bar chart shows the proportion of different clusters within each group.

In the previous analysis, we found that there were also significant differences in several T cell ratios (**Figure 3F**), here we analysed the 6928 cells of the T cell subgroup of **Figure 5A** by principal component analysis and selected the top 10 principal components. Neighbourhood relationships between cells were then calculated at a resolution of 0.3, followed by downscaling and projection using UMAP. UMAP downscaling showed that these cells were classified into 9 clusters (**Supplementary Figure 3A**). Combined with the expression of Cd4, Cd8a, Ccr7, S100a4 (**Supplementary Figure 3B**), we divided the T cell subgroups into two main subgroups CD4^+^T (Cluster0, Cluster2, Cluster6, Cluster7, Cluster8), CD8^+^T (Cluster1, Cluster3, Cluster4, Cluster5). Finally, 3735 CD4^+^ T cells were obtained, and after principal component analysis, the top 10 principal components were selected. Then the neighbour relationship between the cells was calculated at a resolution of 0.4, and then UMAP was used for downscaling and projection. UMAP downscaling showed that these cells were divided into 8 clusters (**Figure 7A**). Also, we observed whether these cells were from sham or from Ischemic stroke (**Figure 7B**). We previously found that the proportion of T cells CD4 memory resting cells increased, and we combined with the Cd44 fractionation (**Figure 7C**) to find that Cluster1 was T cells CD4 memory resting, and we found that subgroup 5 of T cells CD4 memory resting was significantly higher in the Ischemic stroke than in the sham-operated group, based on the distribution of cell proportions (**Figure 7E**). T cells CD4 memory resting in cluster 5 was significantly higher in the Ischemic stroke than in the sham-operated group (**Supplementary Figures 3C, D**). Similarly, we observed the IS-RSGs gene set score in CD4^+^T cells, and the expression of subgroup 5 was lower in T cells CD4 memory resting (**Figure 7D**). CD8^+^T consisted of 3193 cells. After principal component analysis, the top 10 principal components were selected. Neighbourhood relationships between cells were then calculated at a resolution of 0.3, followed by downscaling and projection using UMAP.UMAP downscaling showed that these cells were classified into 7 clusters. (**Figure 7F**). Among them subclusters 1, 2, 3 were increased in Ischemic stroke compared to the sham group (**Figure 7I**), combined with **Figures 7G, H** the three subclusters increased subclusters 1 and 3 were probably CD8(TRLs) cells, where the IS-RSGs gene set score in the subclusters was not seen to be significantly reduced (**Supplementary Figure 3D**).

### Expression specificity analysis of hub genes in microglia cells

**Figure 8:**
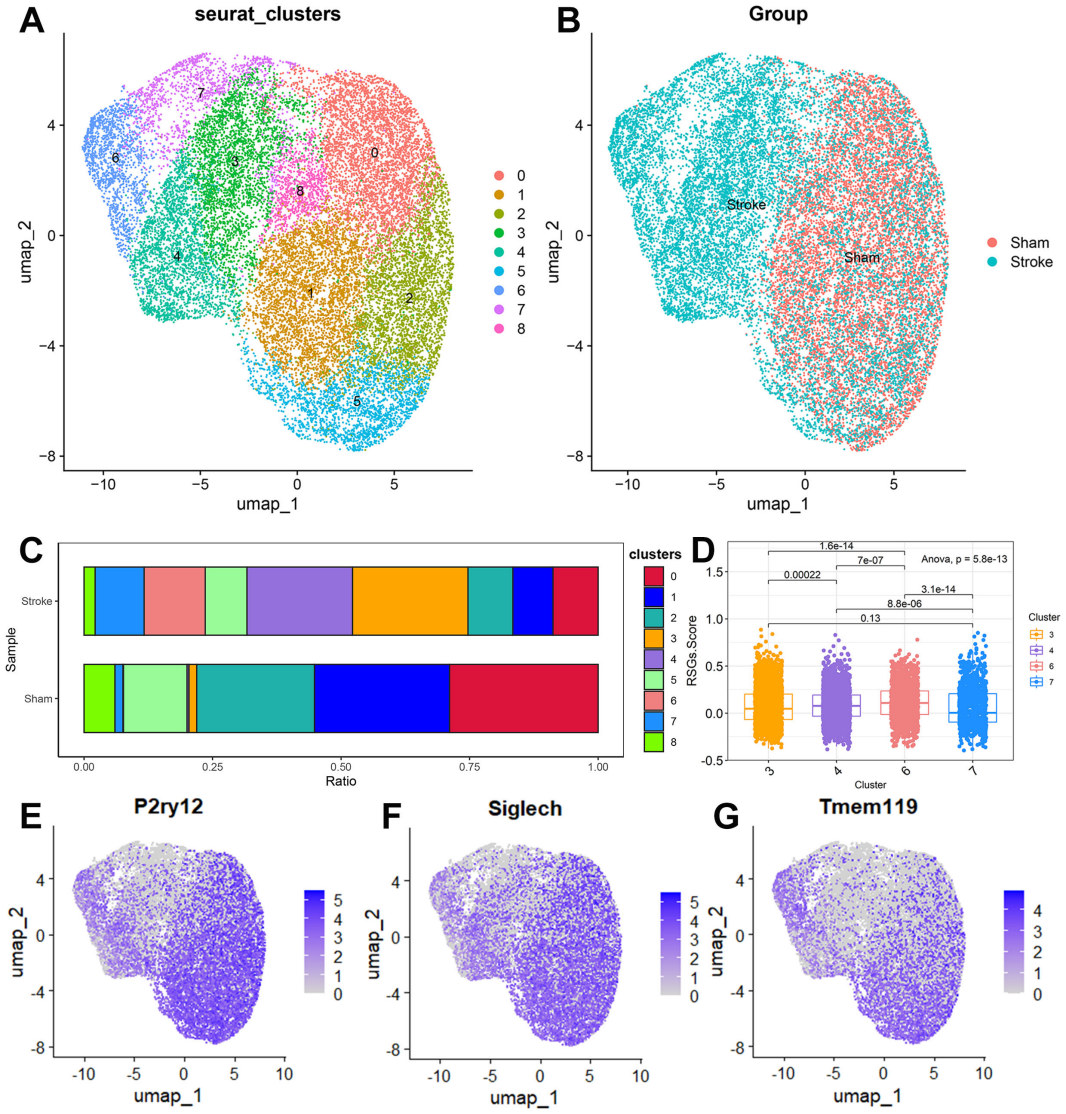
The expression specificity analysis of hub genes in Microglia cells. **(A)** Dimensionality reduction map of Microglia cell subsets. **(B)** Group display of Microglia cell subpopulation type Reduction map (UMAP). **(C)** Stacked bar chart shows the proportion of different clusters within each group. **(D)** The boxplot shows the representation of RSGs Score in the four Clusters. **(E)** UMAP shows the annotation of P2ry12 in Microglia cells. **(F)** UMAP shows the annotation of Siglech in Microglia cells. **(G)** UMAP shows the annotation of Tmem119 in Microglia cells.

Microglia showed different functional states at different stages after stroke. We obtained 50,773 cells from single cells of mouse brain tissue after filtration, and then selected the first 5 principal components through principal component analysis, calculated the neighbourhood relationship between cells with a resolution of 0.8, and then used UMAP for downscaling and projection. UMAP reduction showed that these cells were divided into 20 clusters (**Supplementary Figure 4A**), and we classified the subpopulations by markers specific to each subpopulation, and eventually we divided these 20 subpopulations into 9 cell types (**Supplementary Figures 4B, C**). We further selected 22,241 microglia, selected the first 5 principal components by principal component analysis, calculated the neighbourhood relationships between the cells at a resolution of 0.5, and then reduced and projected them using UMAP. UMAP showed that the cells were divided into 9 clusters. (**Figure 8A**) We found an increase in microglia in clusters 3, 4, 6, and 7 in the ischemic stroke group compared to the sham surgery group (**Figures 8B, C**). The IS-RSGs scores of Cluster3 and Cluster7 in microglia were significantly lower than those of Cluster4 and Cluster6 (**Figure 8D**). We also found that the expressions of P2ry12, Siglech and Tmem119 were lower in Cluster3 and Cluster7 than in other clusters (**Figures 8E–G**).

### Expression of serum RSG protein in patients with ischemic stroke and presentation of patient baseline data

**Figure 9:**
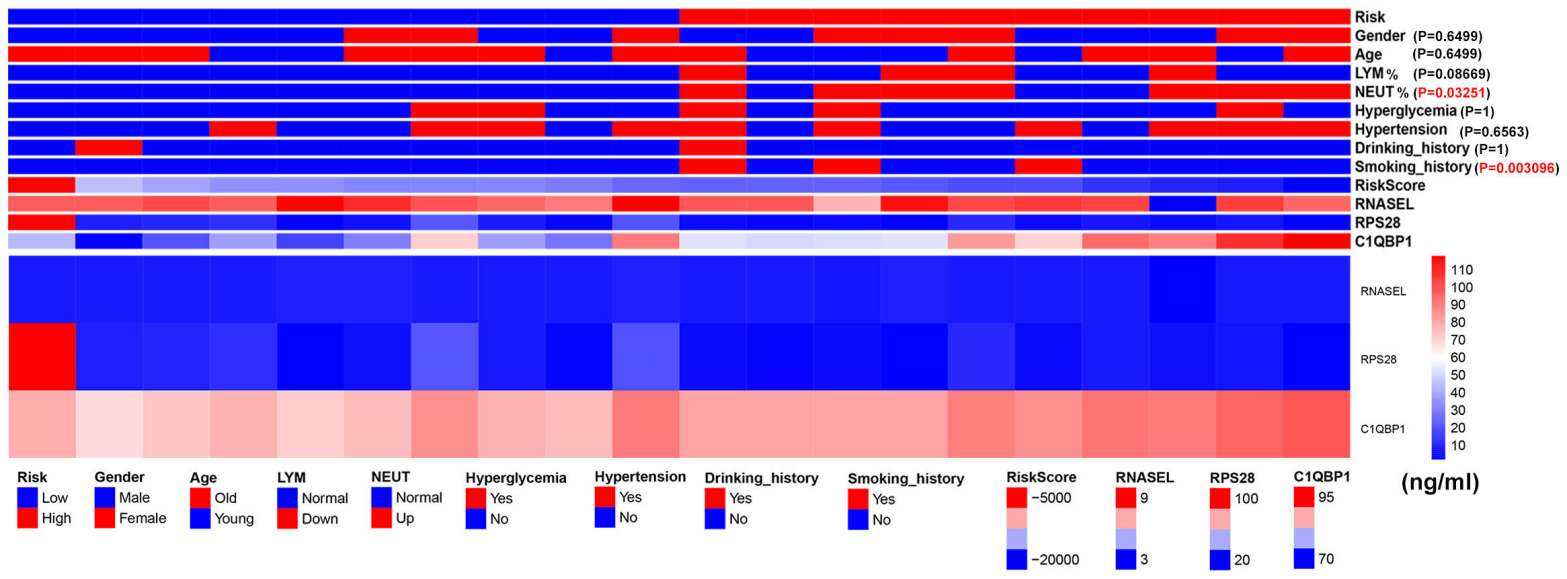
Expression of serum RSG protein in patients with ischemic stroke and presentation of patient baseline data.

We collected serum from 20 stroke patients, and serum ELISA results showed RNASEL, RPS28andC1QBP1.Patients were divided into high and low risk groups according to our scoring method. We analysed the gender, age, hypertension, hyperglycemia, neutrophil ratio, lymphocyte ratio, smoking history and drinking history of these 20 patients. The results showed that neutrophil ratio and smoking history were different between the high and low risk groups (**Figure 9**).

### Screening of relevant small molecule drugs

**Figure 10:**
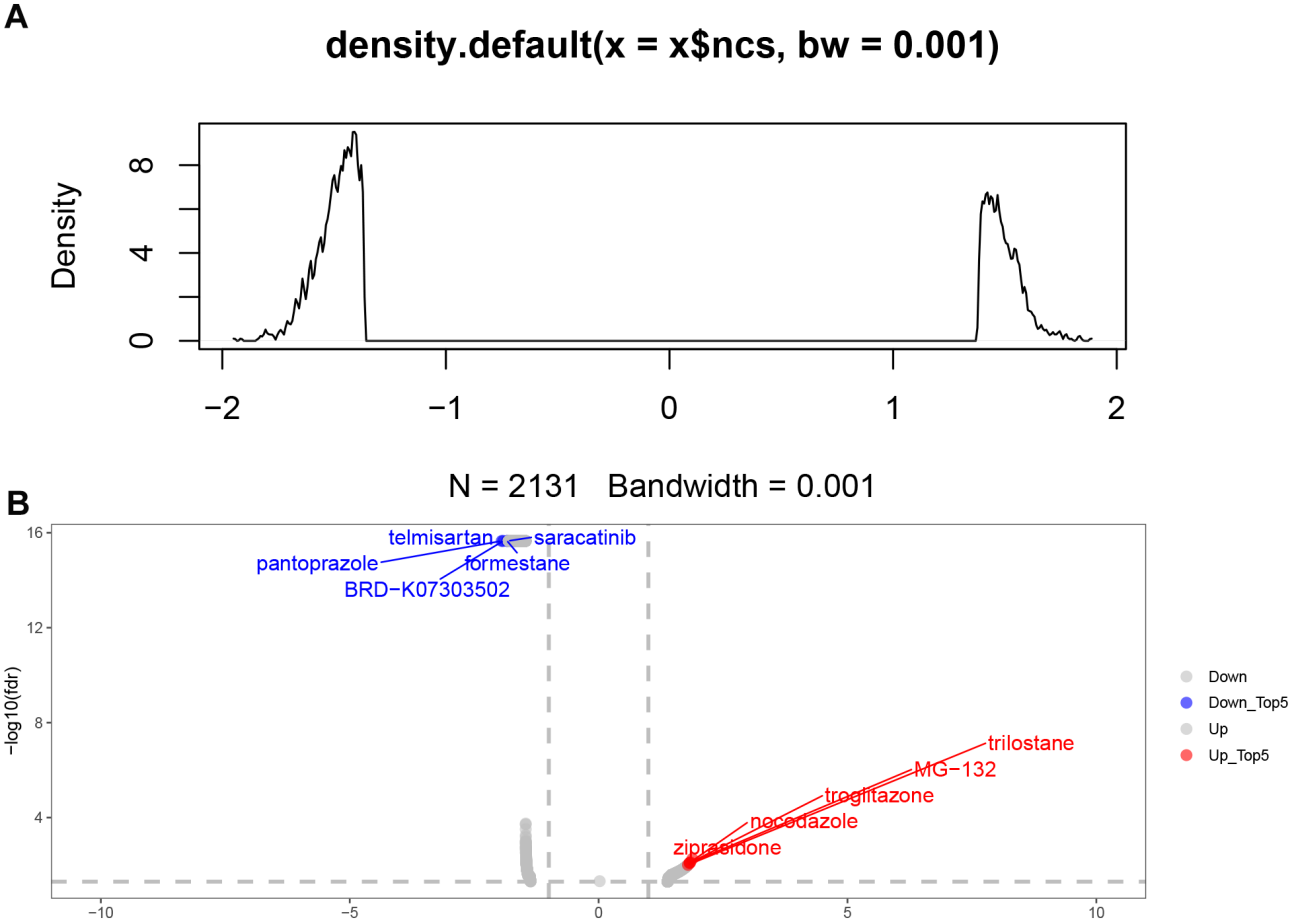
Screening of relevant small molecule drugs. **(A)** 2131 related small molecule drugs were screened. **(B)** The top and bottom 5 small molecule drugs were displayed.

We predicted the differentially expressed genes between RSGs genes combined with RiskTypes via the CMAp (https://clue.io/query) website. After analysing and screening for 2131 relevant small molecule drugs (**Figure 10A**). We presented the small molecule drugs with the top 5 inhibition rankings and the top 5 promotion rankings (**Figure 10B**).

## Discussion

Stroke is the second leading cause of death and a major cause of disability worldwide (29). Rehabilitation for stroke can minimize the impact of disability on normal life, and early diagnosis and effective treatment of ischemic stroke are key to improving clinical outcomes (30, 31). Therefore, our study established a link between ribosome biogenesis genes and the pathogenesis of ischemic stroke. We identified potential IS-RSGs and explored future therapeutic targets via data analysis, thereby facilitating the development of more effective therapeutic strategies and novel drugs against potential therapeutic targets in ischemic stroke.

In our study, we finalized the set of 12 ribosome biogenesis-related genes (EXOSC5, MRPS11, MRPS7, RNASEL, RPF1, RPS28, C1QBP, GAR1, GRWD1, PELP1, UTP, ERI3). These sets of ribosome biogenesis-associated genes play a key role in stroke pathogenesis. These sets of ribosome biogenesis-associated genes play a key role in stroke pathogenesis. Among them, Exosc5 is a component of the RNA exosome complex and is involved in many cellular processes related to RNA processing and degradation. Importantly, mutations in the EXOSC5 gene are associated with an increased risk of sudden cardiac death (32). In a previous study, EXOSC5 was suggested to play an important role in stroke (33). MRPS11 and MRPS7 are members of the mitochondrial ribosomal proteins (MRPs) family, which is essential for the structural and functional integrity of the mitochondrial ribosomal complex (34). It has been shown that MRPS11 shows significant downregulation in the peripheral blood of ischemic stroke patients (35). It has been reported that RNASEL plays an important role in the development of stroke (36, 37), and RNASE also has the functions of regulating the cell cycle (38) and apoptosis (39). Our analysis of single-cell data found that neutrophils increased in the ischemic stroke group. Neutrophils are the first cells to enter the brain after stroke, and they aggravate brain damage through a variety of mechanisms (40). Neutrophil extracellular traps (NETs) that can be released by neutrophils have been reported to be associated with poorer brain injury and stroke outcomes by impairs revascularization and vascular remodelling (41–43). This is consistent with the increase in Cluster0, Cluster1, and Cluster5 in the neutrophil subsets we found in the ischemic stroke group. The RSGs Score of Cluater1 is significantly lower than that of Cluater0 and Cluater5. This subgroup may be specific to the high-risk group we have identified. Microglia show different functional states at different stages after stroke. In our study, two groups of microglia were found. They also showed low expression of homeostasis related genes P2ry12 (44), Siglech (45) and Tmem119 (46) with low ribosome-related gene set scores. Microglia P2ry12 form special somatic junctions where ATP is released from the neuron’s cell body, allowing continuous contact and monitoring of the neuron. After ischemia, the connectome area of microglia covering neurons increases, and the degree of neuronal damage can be reduced by regulating the concentration of calcium ions in neurons and enhancing the activity of mitochondria (47). Decreased P2ry12 expression may affect this function. This is consistent with the hypothesis that we found that the high-risk group had a poor prognosis. Studies have reported that By predicting small molecule drugs through CMAp, we found rilostane, MG-132, troglitazone, nocodazole and ziprasidone, the top 5 up drugs. It has been reported that Nocodazole binds to beta-tubulin and disrupts microtubule assembly/disassembly dynamics, thereby preventing mitosis and inducing tumour cell apoptosis (48). MG-132 effectively blocks the proteolytic activity of 26S proteasome complex. MG-132 is a peptide aldehyde and an autophagy activator. MG-132 can also induce apoptosis (49, 50). These small molecule drugs are expected to improve outcomes in high-risk patients.

In summary, by using mass transcription and single-cell transcription techniques, we revealed the association between Ribosome biogenesis genes and infiltrating immune cells, and selected the features based on 12 Ribosome biogenesis genes as the best machine learning model for constructing a high and low risk group score for grouping patients with moderate scores. The expression of immune cells was different between the high-risk group and the low-risk group. At the single-cell level, we found that the proportion of neutrophils increased, and the score of IS-RSGs related gene set significantly decreased in neutrophils, while the neutrophils subsets of the high-risk group cluster0 and cluster5 were significantly increased in the analysis of neutrophils separately. In microglia, we found that the scores of IS-RSGs related gene sets in cluster3 and cluster7 were significantly reduced. At the stroke patient level, we found that patients in the high-risk group had a higher proportion of neutrophils and more patients with a history of smoking. Our findings reveal the role of Ribosome biogenesis genes in the progression of ischemic stroke and provide new insights into the underlying pathogenic processes and therapeutic strategies for ischemic stroke.

## Limitations and outlook

The limitation of this study was that it was not possible to collect brain tissue of ischemic stroke patients for single-cell sequencing, and only relevant data of mouse brain tissue could be obtained through mouse models for verification. The mechanisms of how the 13 genes we identified function require further research, and we need to collect more samples to explore the differences between high and low risk patients. This study is based on the characteristics of 12 ribosome biogenetic genes as the optimal machine learning model for constructing high - and low-risk group scores. The high-risk group is expected to improve patient outcomes by increasing ribosome-related gene activity.

## Data Availability

The RNA-seq gene expression files was obtained from Gene Expression Omnibus (GEO) database, which can be accessed at (GEO, https://www.ncbi.nlm.nih.gov/geo/) GSE58294 (25)(containing 20 Control and 69 Stroke samples), GSE22255 (26)(containing 20 Control and 20 Stroke samples) GSE16561 (27)(containing 24 Control and 39 Stroke samples).
