# Supplementary material for: Identification of ribosome biogenesis genes and subgroups in ischaemic stroke
Source: Front Immunol. 2024 Sep 2;15:1449158. doi: 10.3389/fimmu.2024.1449158 (PMC11406505; doi:10.3389/fimmu.2024.1449158)
Supplement: Supplementary file 1 [file DataSheet1.docx]

Supplementary Material

# Supplementary Figures and Tables

## Supplementary Figures


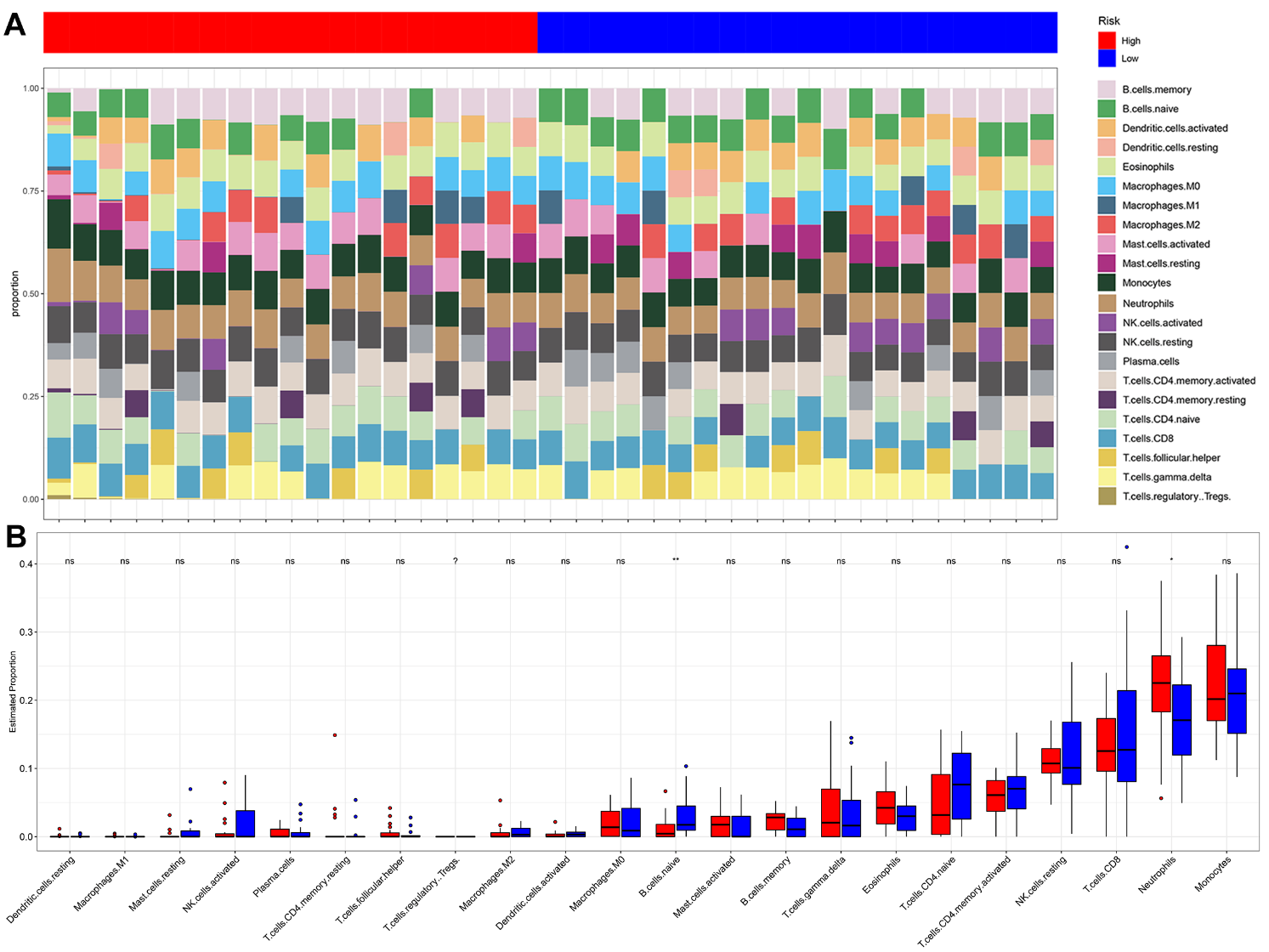


**Supplementary Figure 1.** (A) The relative content of 22 kinds of immune cells high risk stroke patient group and low risk stroke patient group were showed in the histograms.(B) The BarPlot illustrated the difference in immune cell inﬁltration between high risk stroke patient group and low risk stroke patient group.


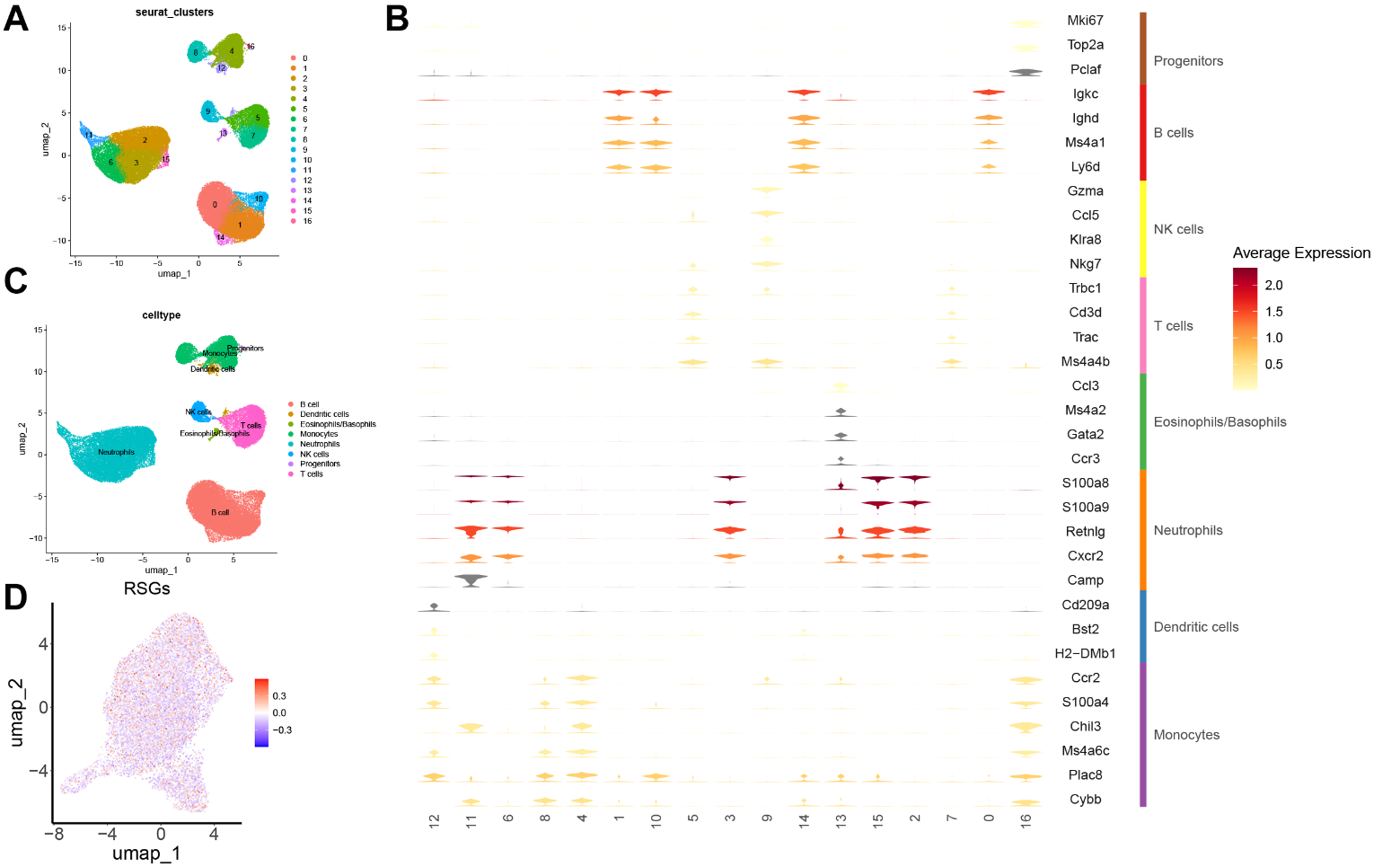
**Supplementary Figure 2.** (A)Cell clusters Reduction Map Display (UMAP). (B) Violin map of marker genes in different cell clusters. (C)Cell types Reduction Map Display (UMAP). (D) UMAP showed the expression of ribosome biogenesis-related genes set in the Neutrophil cells subset of patients.


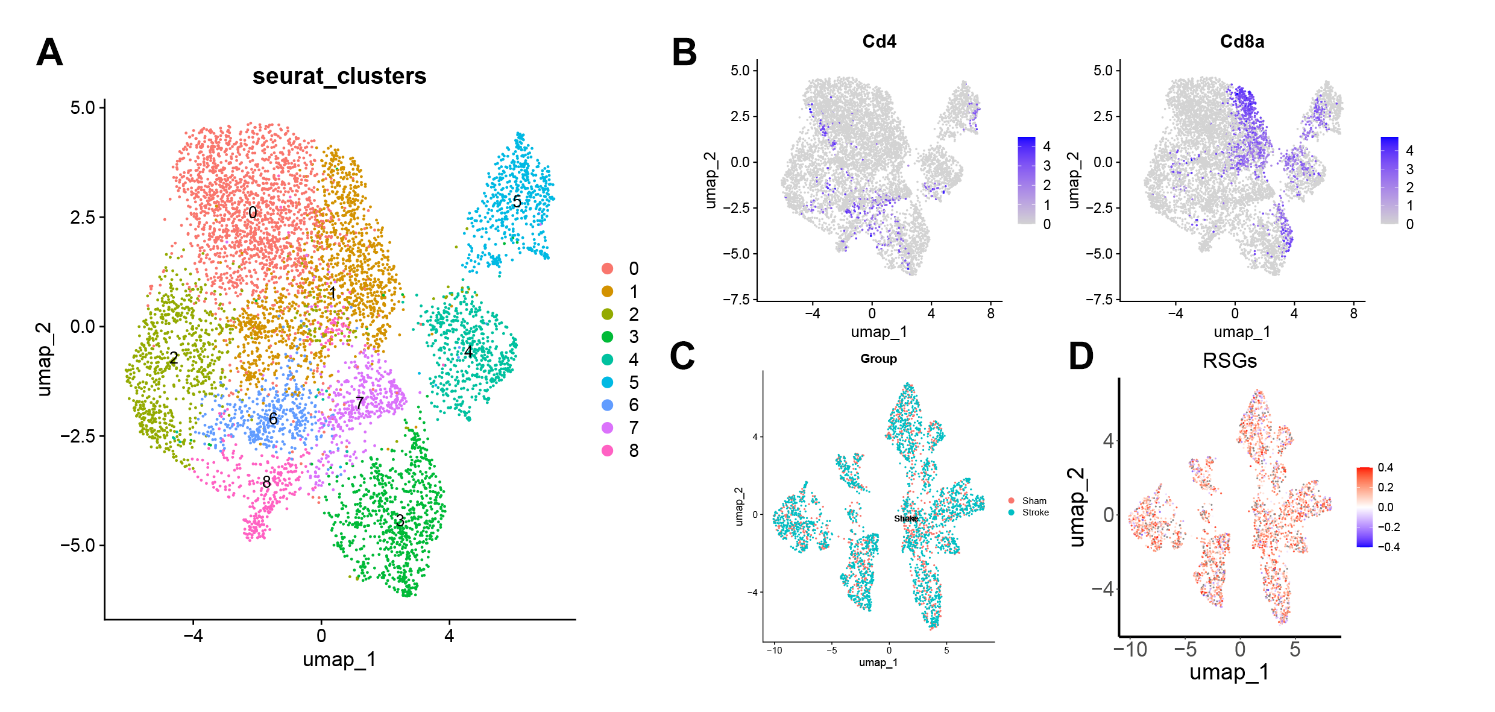


**Supplementary Figure 3.** (A)Dimensionality reduction map of T cell subsets. (B) UMAP shows the annotation of Cd4, Cd8a in T cells. (C) Group display of CD8 T cell subpopulation type Reduction map (UMAP). (D)UMAP showed the expression of ribosome biogenesis-related genes set in the CD8 T cells subset of patients.


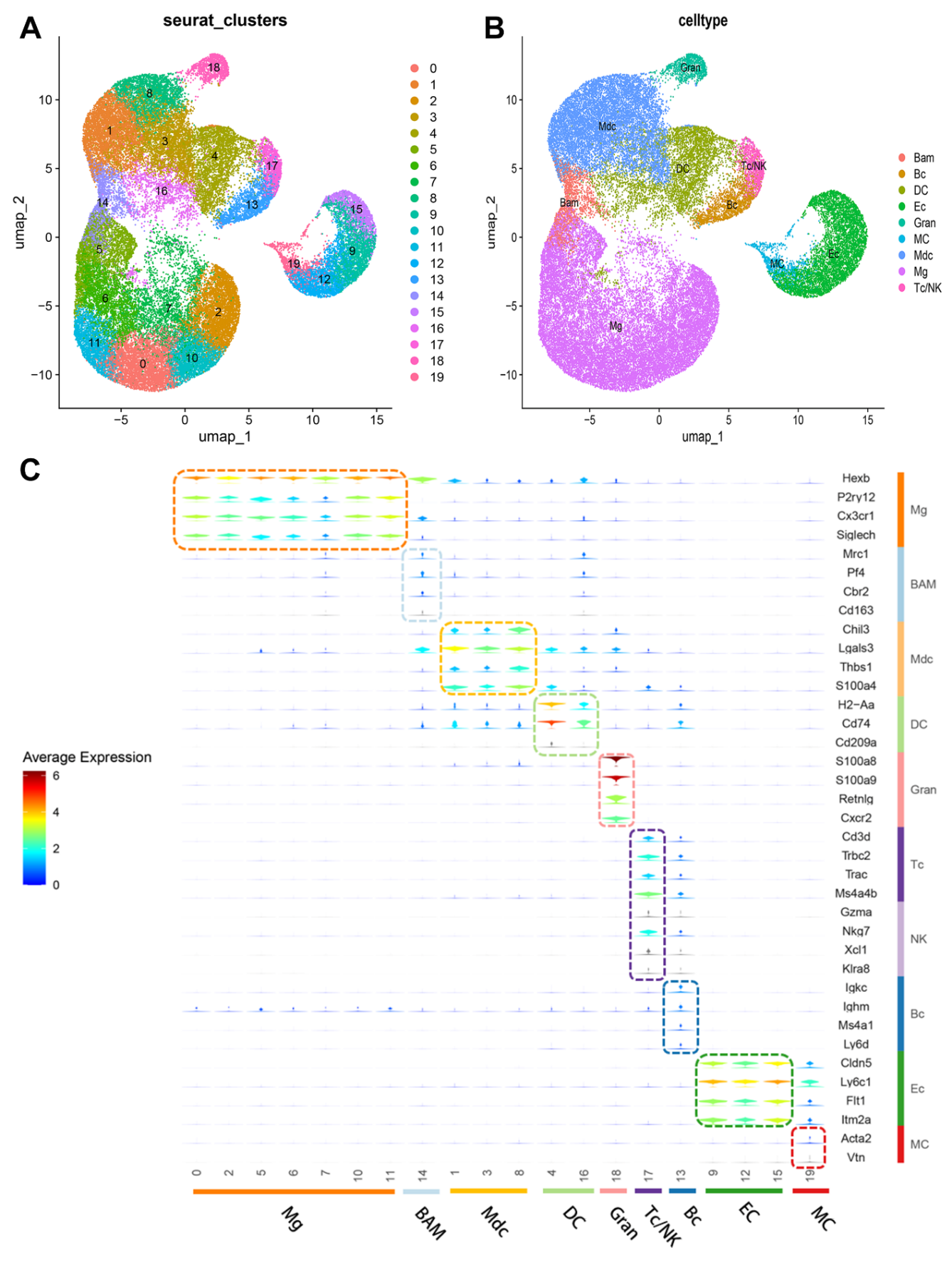


**Supplementary Figure 4.** (A)Cell clusters Reduction Map Display (UMAP). (B)Cell types Reduction Map Display (UMAP). (C) Violin map of marker genes in different cell clusters.

## Supplementary Table

| ID | coef |
| --- | --- |
| C1QBP | -263.250126614001 |
| ERI3 | -148.266156226956 |
| EXOSC5 | -168.111224358709 |
| GAR1 | -80.8009021048234 |
| GRWD1 | -135.456942548514 |
| MRPS11 | -46.2629252208425 |
| MRPS7 | -63.1837251913843 |
| PELP1 | -302.84872796944 |
| RNASEL | 97.0611532888223 |
| RPF1 | -112.443119332536 |
| RPS28 | 165.002466930396 |
| UTP3 | -92.6550657944475 |

**Supplementary Table1** **Model coef**
